# Quantifying the Influence of Factors on the Accuracy of Speech Perception in Mandarin-Speaking Cochlear Implant Patients

**DOI:** 10.3390/jcm12030821

**Published:** 2023-01-19

**Authors:** Xiang Mao, Ziyue Zhang, Yu Chen, Yue Wang, Yijing Yang, Mei Wei, Yao Liu, Yuanxu Ma, Peng Lin, Wei Wang

**Affiliations:** 1 Department of Otorhinolaryngology Head and Neck Surgery, Tianjin First Central Hospital, No. 24 Fukang Road, Nankai District, Tianjin 300192, China; 2 Institute of Otolaryngology of Tianjin, Tianjin 300192, China; 3 Key Laboratory of Auditory Speech and Balance Medicine, Tianjin 300192, China; 4 Key Medical Discipline of Tianjin (Otolaryngology), Tianjin 300192, China; 5 Otolaryngology Clinical Quality Control Centre, Tianjin 300192, China

**Keywords:** cochlear implant, aided hearing threshold, mandarin speech perception, influencing factors, functional relationship

## Abstract

Rehabilitation of hearing perception in cochlear implant (CI) patients is a challenging process. A comprehensive analysis of the characteristics of hearing rehabilitation in Mandarin-speaking CI patients was conducted. We measured the aided hearing threshold (AHT) and the speech perception accuracy (SPA) and collected clinical data. A total of 49 CI patients were included. Significant linear relationships existed between the AHT and SPA. The SPA increased by about 5–7% when the AHT decreased by 5 dB. An apparent individual difference in the SPA was observed under the same AHT, which in some patients was lower than the reference value fitted by the regression model. The timing of both of cochlear implantation and rehabilitation training was found to lead to significant improvement in SPA. The SPA increases by 2.1–3.6% per year of cochlear implantation and 0.7–1.5% per year of rehabilitation training. The time of auditory deprivation can significantly reduce the SPA by about 1.0–1.6% per year. The SPA was still poor in some CI patients when the hearing compensation seemed satisfying. Early cochlear implantation and post-operative rehabilitation are essential for recovery of the patient’s SPA if the indications for cochlear implantation are met.

## 1. Introduction

Cochlear implantation is the most effective way to help patients with severe or extremely severe sensorineural hearing loss (SNHL) reconstruct their hearing function. However, “hearing” is not equal to “understanding”, and cochlear implant (CI) patients may still find it difficult to recover to the same level of hearing perception as ordinary people [1,2,3]. Hearing perception not only requires a complete auditory pathway from the cochlea to the cortex, consisting of the cochlea, auditory nerve, and nuclei in the brain stem and midbrain that transmit sound signals, but also requires a higher level of auditory structures and functions, namely the temporal, parietal, and frontal lobes of the cerebral cortex, to process hearing information [4,5]. Therefore, numerous factors such as characteristics of hearing loss, the cochlear device itself, and pre- or post-operative treatment and rehabilitation may directly or indirectly impact this complex auditory system [6,7,8,9,10]. The issue of hearing rehabilitation after cochlear implantation still lacks a unified conclusion.

With the emergence of government aid policy and corporate social responsibility funding, the number of CI surgeries in China has increased. Many studies have been conducted in recent years targeting the hearing rehabilitation issue of CI patients and revealing deficits in Mandarin speech perception and pronunciation. After summarizing the research, we found that some knowledge gaps still need to be filled. First, some Western scholars have described that the improvement of surgical techniques and the upgrading and iteration of the cochlear device make hearing quality different from the past [11]; thus, the previous research results are no longer applicable today. Recent studies by Chinese scholars have also found a similar phenomenon in which Mandarin-speaking CI patients do not show obvious defects in their perception of tones, which is inconsistent with most earlier studies that believed that CI patients have a weak perception of Chinese language tones [12]. Second, when compared with Western developed countries, the development of Chinese speech audiometry has long been restricted by the lack of audiometric materials and nonstandard audiometric methods, making speech audiometry results measured in previous studies less referential [13]. In recent years, some scholars, such as Professors Lina Huang, Hua Zhang, Fei Ji, and Xin Xi, compiled standardized speech audiometry materials, making significant progress in Chinese Mandarin-speaking speech audiometry in China [14]. For example, the “Xin Ai Fei Yang” Chinese speech audiometric system developed by the PLA General Hospital can be used to perform recognition rate/threshold tests of monosyllabic words, spondaic words, and sentences under quiet/noise conditions, providing an effective speech audiometric method for clinical and scientific research work [15]. Last but not the least, some common factors such as hearing deprivation, residual hearing, and implantation age have been widely considered to have significant effects on speech perception [16,17]. However, few studies have further quantitatively analyzed the influence of such factors. Furthermore, the influence of the CI hearing aid effect on hearing perception has not seriously been considered. It has been proven that in a certain range, increasing the speech intensity level could lead to an improved speech recognition rate in the normal hearing population [18]. It is unknown whether this is fit for CI patients, e.g., reducing the aided hearing threshold (AHT) to improve speech perception accuracy (SPA).

Therefore, a comprehensive and in-depth analysis of the characteristics of hearing rehabilitation in CI patients was conducted. We measured the AHT and the SPA and collected clinical data on hearing loss and pre- or post-operative treatment and rehabilitation. We aimed to reveal the hearing aid condition after cochlear implantation and the quantitative relationships between the AHT and SPA. Finally, we used multiple linear regression models to calculate the functional relationship between the accuracy of consonants, vowels, tones, sentence perception, and related influencing factors after controlling for the effects of AHT. The results can provide support for clinicians to improve treatment methods and guide patients to recover.

## 2. Materials and Methods

### 2.1. Study Participants and Questionnaire

From August 2020 to April 2022, CI patients who were admitted to the Department of Otorhinolaryngology—Head and Neck Surgery, Tianjin First Central Hospital, were recruited. These patients fit the indications for cochlear implantation and had undergone cochlear implantation surgery in our department [19]. The age of inclusion was defined as 5 to 80 years old. Patients with severe cochlear malformation, auditory nerve dysplasia, auditory neuropathy, epilepsy, autism, attention deficit hyperactivity disorder, and other cognitive impairments were excluded. Before the test, medical staff members explained the purpose of the study, test content, data use, privacy protection, and other research details to the patients and their family members. After obtaining consent from the patients or their family members, they signed the informed consent form before data collection.

Eligible participants completed a structured questionnaire concerning socio-demographics, course of hearing loss, surgery, and post-operative rehabilitation with the help of medical staff.

### 2.2. Ethics Statement

This study complied with the Declaration of Helsinki in that the Medical Ethics Committee of Tianjin First Central Hospital approved the research protocol. The review number is 2020N114KY.

### 2.3. AHT and SPA Test

Before collecting the results from the AHT and SPA tests, the audiologist used an otoscope to check the ear canal for cerumen and foreign matters and checked the working condition of the hearing aid (HA) or cochlea. The audiologist also needed to adjust the parameters of the HA or the cochlea according to the patient’s AHT test results and complaints to achieve the appropriate amount of stimulation.

#### 2.3.1. AHT Test

The test was conducted in a soundproof room with a background noise of less than 30 dB (A). The subjects were seated in the middle of the room. The intersection point of the connecting line between the center of both ears and the midline of the room was used as the reference test point. The center point of the connecting line between the two loudspeakers was 1 m from the reference test point with a 45° angle of incidence and at the same height. The test sound signal was selected as a continuous warble tone, and the test began according to the frequency sequence of 1000, 2000, 4000, 1000, 500, and 250 Hz. Subjects were required to cooperate with the audiologist to complete the test and raise their hands immediately after hearing the sound. The audiologist sent a signal 20 dB below the audible hearing level, and if no response was detected, this was incremented at 5 dB until a reaction was generated. The AHT of the frequency was defined as a certain intensity that could be responded to at least twice in the three occurrences of the stimuli. The hearing threshold test for the remaining frequencies was performed according to the above steps.

#### 2.3.2. SPA Test

The test software was the “Xin Ai Fei Yang” Chinese speech testing platform, and the signal intensity was 70 dB SPL. The test contents used in this study were monosyllabic words and sentences under quiet conditions. Each test contained 25 syllables and 10 sentences with 50 keywords. Subjects were required to repeat what they heard immediately after hearing the monosyllabic words and sentences released by the loudspeaker. Monosyllabic words were scored by consonant, vowel, and tone. Sentences under quiet conditions were scored by the keywords contained in the sentence. The audiologist selected “true” or “false” in the testing platform based on the repetition of the subjects. The system calculated the correct rate of consonant, vowel, tone, and quiet sentences.

### 2.4. Data Entry, Sorting, and Statistical Analysis

Questionnaire data collected through the online data collection software could be directly converted into a structured database. The AHT and SPA test results were input through Epidata 3.1 (Epidata Association, Inc., Odense, Denmark) software and were generated in the results database. To meet the statistical analysis requirements of this study, some variables were sorted out and defined. First, the frequencies of 0.5, 1, and 2 kHz accounted for 70% of language intelligibility. The World Health Organization (WHO) issued a classification standard in 1997, adding another hearing threshold of 4 kHz to fully consider the higher-frequency hearing loss of deaf people. Therefore, in this study, the average AHT of these four frequencies were taken as the AHT of the ear side (in dB). Second, the “Xin Ai Fei Yang” Chinese speech audiometric platform was used to test the perception accuracy rate or threshold of monosyllabic words, spondee words, sentences under quiet conditions, and sentences under noise. To improve the testing efficiency, this study only tested the perception accuracy rate of monosyllabic words and quiet sentences. The perception accuracy rates of monosyllabic words were divided into three dimensions for statistical analysis: (1) consonants, (2) vowels, and (3) tones. Third, the subjects were divided into unilateral and bilateral CI patients. Unilateral CI patients were subdivided into CI with HA dual-mode and wearing only a CI. When sorting out the data, the AHT and SPA of the subjects were divided into the cochlear and HA sides for statistical calculation, while for the bilateral cochlea, the average value of the AHT and SPA of both sides was used for statistical calculation. Finally, to calculate the quantitative relationship between SPA and potential influencing factors, this study selected four continuous variables to include in linear regression models: (1) time of auditory deprivation, (2) age of cochlear implantation, (3) time of cochlear implantation, and (4) time of rehabilitation training. Time of auditory deprivation was defined as the time interval (weeks) during which the patient started to feel conscious hearing loss on the side of the ear with the cochlear implantation. The time of rehabilitation training (hours) was defined as the duration (weeks) of rehabilitation training after the cochlear implantation multiplied by the average training times per week and multiplied by the average training time (hours) of each training. If the subject was still performing rehabilitation training when participating in this study, the training deadline was defined as the survey date to calculate the duration (weeks).

A one-way analysis of variance (ANOVA) was used to compare the differences in AHT of different frequencies and the differences in SPA on different speech dimensions. An independent sample *t*-test was used to compare the difference in AHT and SPA between the cochlear side and the HA side. The linear relationships between SPA of different speech dimensions and AHT were fitted by unary linear regression, and regression equations, correlation test results, and the goodness of fit (*R*^2^) were considered output. Multivariate linear regression was used to calculate the effect of time of auditory deprivation, age of cochlear implantation, time of cochlear implantation, and time of rehabilitation training on perception accuracy of consonant, vowel, and tone of monosyllabic words and sentences under quiet conditions. The variance inflation factor (VIF) was collinearity diagnostic. This multivariate linear regression adopted a three-level model correction method: (1) model 1: the SPA was used as the dependent variable, and time of auditory deprivation, age of cochlear implantation, time of cochlear implantation, and time of rehabilitation training were defined as the independent variables, and linear regression analysis was performed; (2) model 2: AHT was adjusted and added to model 1; and (3) model 3: age was adjusted and added to model 2. Because of the significant collinearity between age and age of cochlear implantation (variance inflation factor (VIF) > 10), the linear regression calculation of the age of cochlear implantation and the SPA was not determined in model 3. IBM SPSS Statistics 20.0 (IBM Inc.) was used for the statistical analysis, and *p* < 0.05 was defined as a statistical difference. Graphpad Prism 5 (Graph Pad Software Inc.) was used to construct forest maps and scatter maps.

## 3. Results

### 3.1. Clinical Characteristics of the Subjects

A total of 49 CI patients were included in this study, of which 27 had unilateral cochlear implantation (55.1%), 16 had cochlea with HA dual mode (32.7%), and 6 underwent bilateral cochlear implantation (12.2%). The median age of the subjects was 17.2 (10.7–39.2) years old. SNHL (53.1%) and large vestibular aqueduct syndrome (LVAS) with SNHL (36.7%) were the leading causes of hearing loss. The median duration from self-reported conscious hearing loss to severe hearing loss was 0.0 (0–8.8) years. The median time of auditory deprivation was 3.0 (0.7–7.3) years, and the highest proportion was auditory deprivation within 1 year (36.7%). The median age of cochlear implantation was 13.6 (4.5–39.0) years, and the median time of cochlear implantation was 1.8 (0.1–7.4) years. Thirty-one patients (63.3%) received speech rehabilitation training after cochlear implantation. The median training intensity was 9.0 (3.3–40.0) hours per week, and the median length of the rehabilitation training was 82.7 (24.5–325.1) weeks. Other clinical characteristics of the subjects are shown in Table 1.

### 3.2. Characteristics of AHT and SPA

**Figure 1:**
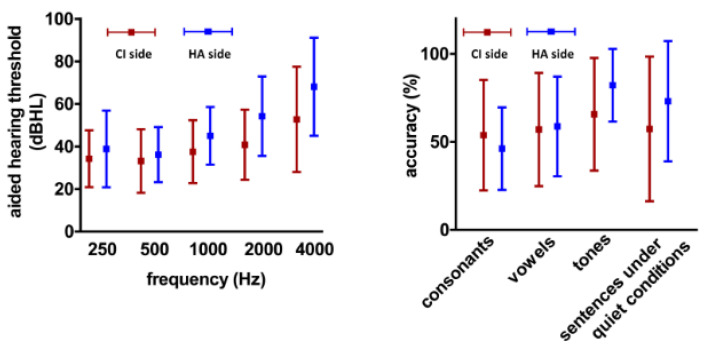
The characteristics of AHT and SPA in cochlear and HA sides of 49 CI subjects. Among the 49 CI patients (unilateral implantation/bilateral implantation/dual mode, cochlea with HA), 55 ear sides wore CI, and 16 ear sides wore HAs.

The AHT and SPA on the HA side of the dual-mode subjects were taken as the control group (16 cases) and compared with those on the cochlear side of all subjects. The average AHT of the cochlear side was significantly lower than that of the HA side (39.7 ± 18.6 dB versus 48.5 ± 20.7 dB, *t* = −3.3; *p* = 0.001). The forest map showed that the AHTs were lower at low frequencies than at high frequencies both on the CI and HA sides, and this characteristic seems more evident on the HA side. The analysis of variance (ANOVA) showed significant differences in AHTs at different frequencies (F_cochlear_ = 11.3, *p* < 0.001; F_HA_ = 7.0; *p* < 0.001), as shown in Figure 1.

The average SPA of consonants, vowels, tones, and sentences in quiet conditions was 58.5% ± 34.4% on the cochlea side and 65.0% ± 29.7% on the HA side. There is no significant difference between the two sides (*t* = −1.4; *p* = 0.169). The ANOVA yielded no significant differences in the SPA of consonants, vowels, tones, and sentences under quiet conditions on the cochlear side (F = 0.9, *p* = 0.445). However, there is a significant difference in those on the HA side (F = 3.6: *p* = 0.011). The accuracy of the consonants perception was the worst (46.2% ± 23.4%), while the accuracy of the perception of the tone was the best (82.2% ± 20.6%), as shown in Figure 1.

### 3.3. The Linear Relationship between AHT and SPA

**Figure 2:**
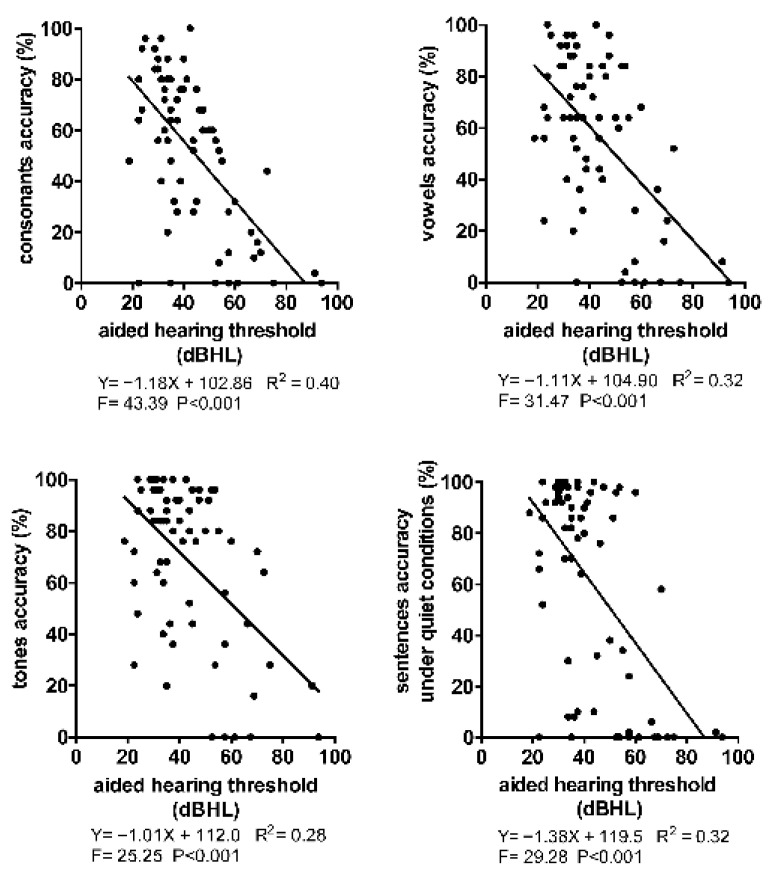
The linear relationship between AHT and SPA in 49 CI subjects. Among the 49 CI subjects, 16 CI with HA dual-mode subjects and 6 bilateral CI subjects were evaluated. Therefore, a total of 71 hearing-aided ears were included for analysis. *R*^2^ was the goodness of fit, and F was the result of the variance test for the regression model. *p* < 0.05 indicates a linear relationship between AHT and SPA.

The AHT and SPA of both ear sides of all subjects were included. It was found that significant linear relationships between the AHT and perception accuracy of consonants, vowels, tones, and sentences under quiet conditions existed (Figure 2). That is, under the condition of not affecting comfort, appropriately increasing the hearing compensation of the HA or CI to reduce the AHT can effectively improve SPA. According to the results calculated by the linear model, the SPA increases by about 5% to 7% when the AHT decreases by 5 dB. However, it can be observed from the scatter plot that under the same AHT, a significant individual difference in the SPA of these subjects was observed. The SPA of some subjects was significantly lower than the reference value under the AHT fitted by the regression model (Figure 2).

**Figure 3:**
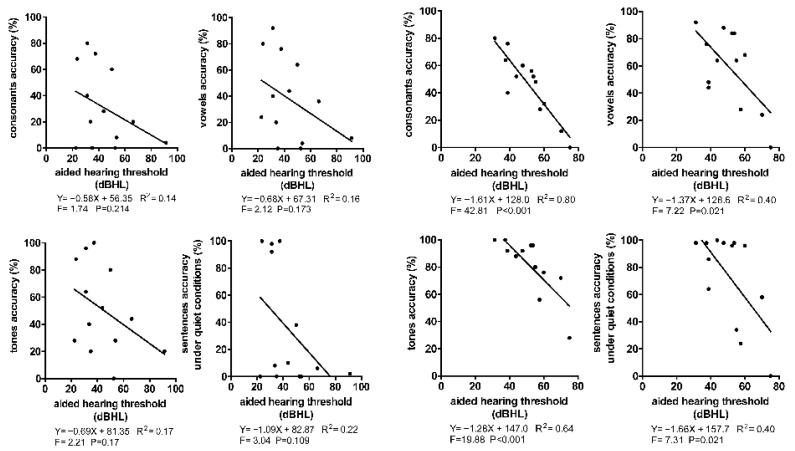
The linear relationship between AHT and SPA in 16 subjects with cochlea and HA dual mode. The left four subparagraphs are the CI side, the right four subparagraphs are the HA side. *R*^2^ represents the goodness of fit, and F was the result of the variance test for the regression model. *p* < 0.05 indicates a linear relationship between AHT and SPA.

To further compare the difference in the linear relationships between the AHT and SPA on the cochlear and HA sides, only 16 dual-mode subjects were selected. The aforementioned linear relationship seemed more evident on the HA side but not on the cochlear side (Figure 3). It can be observed from the scatter plot that under the same AHT, a more significant individual difference in the SPA on the cochlear side when compared with the HA side was observed. On the cochlear side, the SPA of nearly half of the subjects was significantly lower than the reference value under the AHT fitted by the regression model. Especially in the perception accuracy of sentences under quiet conditions, the accuracy rates of most subjects were below 20%, which ignored the influence of AHT (Figure 3). It is suggested that besides the AHT, some pre- or post-operative factors significantly affected SPA on the cochlear side.

### 3.4. Functional Relationship between SPA and Related Influencing Factors

The time of cochlear implantation can significantly improve the perception accuracy of consonants, vowels, tones, and sentences under quiet conditions. Especially after adjusting for AHT and age, the effect is still robust (*p*_sentences_ = 0.057; else *p* < 0.05), *b* = 0.04–0.07; that is, the SPA increases by 0.04% to 0.07% per week after cochlear implantation (2.1–3.6% per year). The time of auditory deprivation can significantly reduce the perception accuracy of consonants, vowels, and tones (*p* < 0.05), *b* = −0.03–(−0.02); that is, the perception accuracy decreases 0.02–0.03% per week of auditory deprivation (about 1.0–1.6% per year). However, the effect on perception accuracy of sentences under quiet conditions was not statistically significant (*p* = 0.132). The time of rehabilitation training could significantly improve the perception accuracy of consonants and vowels (*p* < 0.05) but had no significant effect on tone and sentences under quiet conditions, *b* = 0.001–0.002; that is, the perception accuracy of consonants and vowels increases by 0.001% to 0.002% per hour of rehabilitation training (estimated by 2 h of rehabilitation training every day, about 0.7–1.5% per year). The age of cochlear implantation had no significant effect on the perception accuracy of consonants, vowels, tones, and sentences under quiet conditions (*p* > 0.05 for all). More details shown in Table 2.

## 4. Discussion

Our study found that patients with severe SNHL had an ideal hearing-aid effect through the CI. The CI has an advantage for high-frequency hearing compensation when compared with the HA. A significant linear relationship between AHT and SPA was found. With every 5 dB increment in AHT, SPA increased by about 5% to 7%. However, in some subjects, the AHT and SPA on the cochlear side do not follow this relationship and present as a disproportionate decline in speech perception. The time of cochlear implantation and rehabilitation training can significantly improve SPA. It seems that the time of cochlear implantation is the most effective in improving the SPA of all dimensions (2.1–3.6% per year). The time of auditory deprivation can significantly reduce patients’ SPA by 1.0% to 1.6% per year. Moreover, the accuracy of sentence perception did not appear to be susceptible to auditory deprivation or rehabilitation training.

By comparing the difference in AHT and SPA between the cochlear and the HA sides, two characteristics were found. First, both CI and HA showed that the hearing compensation of low frequencies was better than that of high frequencies, and it seems that the cochlea has better high-frequency hearing compensation when compared with the HA (Figure 1). Although CI and HA exhibit similar characteristics of hearing compensation, the reasons for this similarity are different. On the cochlear side, some patients may not tolerate the stimulation of the electrode array located at the bottom of the cochlea for high-frequency hearing compensation in the initial period after cochlear implantation. Thus, audiologists often do not give enough stimulation when setting the stimulation parameters, which shows the phenomenon of insufficient high-frequency compensation. As the patient’s tolerance to electrode stimulation improves, the audiologists will gradually increase the amount of electrical stimulation of the high-frequency array, and the corresponding high-frequency compensation will improve. On the HA side, because of the anatomical structure of the cochlea, cochlear hair-cell damage in hearing-impaired patients mostly starts from the bottom of the cochlea [20,21]. It is more likely to appear that the inner hair cells at the bottom of the cochlea are severely damaged. Only increasing the high-frequency sound stimulation of the HA may have limited capability to improve the high-frequency compensation in patients or even cause howling and distortion, so the high-frequency AHT on the HA side is difficult to reduce. Second, we found no statistical difference in the perception accuracy of consonants, vowels, tones, and sentences under quiet conditions on the cochlear side. In the past, some scholars believed that tone perception mainly depended on the fine resolution of low and middle frequency. The cochlea could not encode the fine structure of this part of the sound, so the patients had poor tone perception accuracy [22]. However, our study did not find that the accuracy of tone perception on the cochlear side is significantly inferior to that of vowels or consonants. The reason may be that technological generations of speech processors and implantation surgery have effectively improved the resolution of sound and listening satisfaction, compensating for the CI patients’ perception accuracy of tones. A recent study found that the accuracy of tone perception in Mandarin CI recipients was even significantly higher than that of the consonants, vowels, and monosyllabic words [12]. However, the CI recipients in that research had a longer time of cochlear implantation (2.8 versus 1.8 years), which could result in a more complete speech perception recovery. For the HA side, significant differences in the perception accuracy of these four speech dimensions were found. We found that the HA side has a poor perception accuracy of consonants and vowels, which may be because the frequency range of consonants is generally above 3 KHz. Consonants and vowels are always combined to form syllables, and the HA has limited hearing compensation for high-frequency hearing loss; thus, it affects the perception accuracy of the consonants and vowels on the HA side. In conclusion, the results suggest that the CI has advantages for high-frequency hearing compensation that may improve SPA.

We found a significant linear relationship between AHT and SPA. For every 5 dB decrease in the AHT, the SPA increased by 5% to 7% (Figure 2). Previous studies conducted in the normal hearing population have also found a similar phenomenon. That is, a functional relationship between speech intensity level and speech recognition rate and performance–intensity (P–I) function curves were found [18]. However, we found that some patients’ SPA on the CI side did not follow this functional relationship, indicating that the SPA was still poor when the hearing compensation seemed satisfying. Some scholars have found a similar phenomenon in the research of hearing-impaired patients with retro-cochlear diseases such as auditory neuropathy and SNHL. Unlike normal-hearing people, the relationship between speech intensity level and speech recognition rate shows obvious non-monotonicity and dispersion [23,24]. Some scholars call this phenomenon a “disproportionate decline in speech comprehension of hearing loss people” [24]. We formed some of the following conjectures about our results: First, the principles of hearing compensation between HA and cochlea were utterly different. HAs realize hearing compensation by amplifying the sound at specific frequencies, and the main function of an HA is to stimulate the auditory nerve to facilitate hearing by completing acoustic electrical conversion through cochlear inner hair cells. The cochlea bypasses the acoustic electrical conversion process, and the electrodes implanted in the cochlea directly stimulate the auditory nerve with artificially encoded electrical signals to facilitate hearing [25]. This synthetic electrical hearing from the cochlea is more challenging to interpret and requires gradual adaptation by the advanced hearing center of the patients. Second, the hearing threshold reflects the function of the lower level of the auditory pathway, namely auditory nerves, brain stem, and midbrain, while speech perception requires a higher level of auditory function, namely the auditory cortex [4,5]. In clinical practice, the hearing-loss characteristics of the cochlea side must be more severe than that of the HA side since there is still some residual hearing and not complete auditory deprivation. Hence, the degradation of the auditory-related neural structures and functions makes it more challenging for the cochlear side to perceive speech when compared with the HA side even if it has achieved satisfying hearing compensation. It is also called “hearing but not understanding”, which often appears in clinical practice. This phenomenon also suggests that factors other than the cochlear device affect SPA in cochlear sides.

In addition, we found that this phenomenon shows different characteristics of the relationship between the accuracy of sentence perception under quiet conditions and the AHT on the cochlear and HA sides. The scatter plot shows that the increase in AHT at the HA side did not significantly affect the accuracy of sentence perception, and the accuracy of most patients is still close to 100%; however, on the cochlear side, decreasing the AHT did not lead to a significant improvement in the accuracy of sentence perception, and the accuracy of most patients was still less than 20%. Some scholars have previously described the concept of “integration of multiple language cues”. The auditory center will integrate multiple language cues of sentences and combine them with its own auditory experience to conduct a comprehensive perception [26]. Thus, it is possible for hearing-impaired patients to accurately perceive sentences even if they lose the perception ability of some elements in sentences. Our results show that when attributed to the aforementioned “hearing redundancy” relying on the limited perception ability on the elements of sentences, the HA side still produced good sentence perception accuracy despite an increased AHT. However, for the cochlear side, the auditory center’s extremely poor hearing perception ability cannot catch the available elements in sentences transmitted from the cochlea when perceiving sentences, so the accuracy of sentence perception cannot be effectively improved although the AHT decreased.

The available research has proven that many related factors may affect SPA after cochlear implantation [6,7,8,9,10]. Based on previous research, this study further accurately quantified the impact of these factors on speech perception after considering the impact of AHT and age. Based on the multiple linear regression model, we found that the time of cochlear implantation is the only factor that has a significant role in promoting the accuracy of all four dimensions (consonant, vowel, tone, and sentence) in Mandarin Chinese speech perception. It is estimated that after cochlea implantation, SPA increases by 2.1% to 3.6% per year, and the improvement in consonants and vowels was slightly higher than in tone and sentence. When compared with the time of cochlear implantation, the effect of the time of rehabilitation training on speech perception is relatively limited. Our study found that it can significantly improve the perception accuracy of consonants and vowels, while the effect on tone and sentence perception is not apparent. As estimated, after 2 h of daily rehabilitation, these parameters can increase by 0.7% to 1.5% per year. Although the estimated numerical annual improvement seemed small after considering that the speech perception after cochlear implantation is affected by the combined action of these factors (there may be interaction), the calculated annual improvement cannot be simply considered as the actual improvement by the patients. In fact, after cochlear implantation, these patients can directly or indirectly undergo hearing rehabilitation training through communication, work, and learning in daily life. Time of auditory deprivation before cochlear implantation could significantly reduce the perception accuracy of consonants, vowels, and tones, and auditory deprivation for 1 year can reduce perception accuracy by 1.0% to 1.6%. Therefore, if the indications for cochlear implantation are met, cochlear implantation and post-operative rehabilitation training can be carried out as early as possible, which is efficacious for improving speech perception ability. In addition, we found no statistical correlation between the age of cochlear implantation and each dimension of SPA. The reason may be that the hearing-impaired patients included in this study, whether pre-lingual deaf children or post-lingual deaf adults, had short periods of hearing deprivation, and no apparent correlation between the age of cochlear implantation and the time of hearing deprivation was found. We also found that compared with monosyllabic word perception (consonant, vowel, and tone), rehabilitation training and auditory deprivation seemed to have little impact on sentence perception. The time of the cochlear implantation no longer had statistical significance in terms of sentence perception after adjusting the AHT and age (*p* = 0.057). This phenomenon is similar to the aforementioned functional relationship between the AHT and sentence perception accuracy, suggesting that sentence perception requires more complex “multiple language cues integration”. The improvement or reduction in monosyllabic word perception caused by factors such as hearing rehabilitation training and auditory deprivation may not significantly affect the perception accuracy of a whole sentence in a short time, so we deduced that sentence perception might require a higher rehabilitation threshold of hearing.

Although this study is as rigorous as possible from the point of view of the test of AHT and SPA in addition to the collection and estimation of clinical data on hearing loss and pre- or post-operative treatment and rehabilitation, some limitations should still be described. First, as the number of patients with CIs is relatively low in our daily practice, especially the CI with HA dual-mode patients, the number of samples included in this study cannot lead to an unbiased estimation of the characteristics of the entire CI population. Limited sample size is not competent for more sophisticated analysis to reveal the different characteristics among different age populations. However, the available data have already preliminarily revealed speech perception characteristics in CI patients so that an enlarged sample size may yield a more accurate prediction. Second, many factors of hearing loss and pre- or post-operative treatment and rehabilitation affect hearing rehabilitation, and an interaction between different factors amplifying or antagonizing the effect on speech perception may exist. This study only analyses the clinical data we can collect and controls the effect of AHT and age through multivariate linear regression to diminish the confounding effect. Third, the characteristics of hearing loss, the degradation of the hearing center after hearing deprivation, and the restoration of hearing function after cochlear implantation in patients are very complex, dynamic change processes. The functional model cannot accurately fit this process. We showed that factors of hearing loss and pre- or post-operative treatment and rehabilitation might have different levels of effect on hearing rehabilitation and then preliminarily quantified the intensities of these factors. Due to the limited number of samples, this study did not calculate the functional relationship in each subgroup after stratified grouping of the subjects according to such characteristics as age or hearing loss.

## 5. Conclusions

Decreasing AHT could lead to an increase in SPA in CI patients. However, some patients’ speech perception was still poor when the hearing compensation seemed satisfactory. Some factors of hearing loss and pre- or post-operative treatment and rehabilitation other than the cochlear device could significantly affect speech perception. This study quantifies the effect intensity of these factors, suggesting that if the indications for cochlear implantation are met, cochlear implantation and post-operative rehabilitation training can be carried out as early as possible, which is efficacious in improving speech perception.

## Figures and Tables

**Table 1:** The clinical characteristics of 49 CI subjects.

	N	%		N	%
Age (years)			Duration of speech rehabilitation training (years)		
1–10	8	16.3	<0.5	8	27.6
10–20	19	38.8	0.5–1	3	10.3
20–30	6	12.2	1–2	5	17.2
30–40	4	8.2	2–4	4	13.8
40–50	4	8.2	>4	9	31.1
>50	8	16.3	Speech rehabilitation training after cochlear implantation		
The reason for deafness			Yes	31	63.3
SNHL + LVAS	18	36.7	No	18	36.7
Drug-induced deafness	5	10.2	Genes for deafness		
SNHL	26	53.1	Yes	11	22.4
Time of auditory deprivation (years)			No	10	20.4
<1	18	36.7	Unknown	28	57.2
1–2	4	8.2	Hearing aid mode		
2–4	9	18.4	The unilateral cochlea, right side	20	40.8
4–10	7	14.3	The unilateral cochlea, left side	7	14.3
>10	11	22.4	Dual mode, right cochlea with left HA	8	16.3
Time of cochlear implantation (years)			Dual mode, left cochlea with right HA	8	16.3
<0.5	18	36.7	Bilateral cochlea	6	12.3
0.5–1	4	8.2	Bilateral cochlear implantation		
1–7	14	28.6	Bilateral implants were implanted simultaneously	3	50
>7	13	26.5	The right side was implanted first, and the left side was implanted later	1	16.7
Age of cochlear implantation (years)			The left side was implanted first, and the right side was implanted later	2	33.3
<4	10	20.4	Time from self-reported hearing loss to self-reported severe hearing loss (years)		
4–10	9	18.4	Time without interval	24	49
10–20	11	22.4	<5	8	16.3
20–30	12	24.5	5–10	10	20.4
20–31	7	14.3	>10	7	14.3
The intensity of speech rehabilitation training (hours/week)			HA wearing before cochlear implantation		
<3	8	25	No	13	26.5
3–10	10	31.3	Yes	36	73.5
10–20	3	9.4	Daily communication with HAs or lip reading		
20–30	2	6.3	Lip reading	16	44.4
>30	9	28	HAs	20	55.6

SNHL, sensorineural hearing loss; LVAS, large vestibular aqueduct syndrome.

**Table 2:** Multiple linear regression analysis of factors associated with the SPA in CI subjects.

		*R* ^2^	*b*	*t*	*p*
Consonants					
Model 1	Time of auditory deprivation	0.138	−0.027	−2.740	0.009
	Age of cochlear implantation	0.025	−0.232	−1.096	0.279
	Time of cochlear implantation	0.363	0.081	5.173	<0.001
	Time of rehabilitation training	0.166	0.002	3.058	0.004
Model 2	Time of auditory deprivation	0.377	−0.02	−2.339	0.024
	Age of cochlear implantation	0.319	−0.185	−0.185	0.306
	Time of cochlear implantation	0.492	0.063	4.141	<0.001
	Time of rehabilitation training	0.393	0.002	2.617	0.012
Model 3	Time of auditory deprivation	0.365	−0.021	−2.281	0.028
	Time of cochlear implantation	0.481	0.068	3.912	<0.001
	Time of rehabilitation training	0.395	0.002	2.588	0.013
Vowels					
Model 1	Time of auditory deprivation	0.147	−0.028	−2.845	0.007
	Age of cochlear implantation	0.032	−0.265	−1.240	0.221
	Time of cochlear implantation	0.332	0.079	4.834	<0.001
	Time of rehabilitation training	0.133	0.002	2.684	0.010
Model 2	Time of auditory deprivation	0.377	−0.021	−2.456	0.018
	Age of cochlear implantation	0.316	−0.218	−1.201	0.236
	Time of cochlear implantation	0.463	0.06	3.794	<0.001
	Time of rehabilitation training	0.362	0.001	2.193	0.033
Model 3	Time of auditory deprivation	0.374	−0.022	−2.444	0.019
	Time of cochlear implantation	0.435	0.06	3.305	0.002
	Time of rehabilitation training	0.362	0.001	2.087	0.043
Tones					
Model 1	Time of auditory deprivation	0.174	−0.03	−3.146	0.003
	Age of cochlear implantation	0.003	−0.082	−0.382	0.704
	Time of cochlear implantation	0.209	0.062	3.529	0.001
	Time of rehabilitation training	0.073	0.001	1.921	0.061
Model 2	Time of auditory deprivation	0.391	−0.024	−2.795	0.008
	Age of cochlear implantation	0.288	−0.036	−0.195	0.846
	Time of cochlear implantation	0.369	0.042	2.440	0.019
	Time of rehabilitation training	0.314	0.001	1.335	0.188
Model 3	Time of auditory deprivation	0.398	−0.025	−2.920	0.006
	Time of cochlear implantation	0.348	0.041	2.192	0.034
	Time of rehabilitation training	0.321	0.001	1.455	0.152
The sentences under quiet conditions					
Model 1	Time of auditory deprivation	0.098	−0.03	−2.211	0.032
	Age of cochlear implantation	0.004	−0.123	−0.440	0.662
	Time of cochlear implantation	0.179	0.074	3.133	0.003
	Time of rehabilitation training	0.067	0.002	1.795	0.079
Model 2	Time of auditory deprivation	0.414	−0.019	−1.748	0.087
	Age of cochlear implantation	0.374	−0.051	−0.225	0.823
	Time of cochlear implantation	0.422	0.042	1.939	0.059
	Time of rehabilitation training	0.392	0.001	1.172	0.248
Model 3	Time of auditory deprivation	0.392	−0.019	−1.541	0.132
	Time of cochlear implantation	0.413	0.05	1.963	0.057
	Time of rehabilitation training	0.396	0.001	1.256	0.216

The CI side of the 49 subjects were included. For subjects with bilateral CI, the average AHT and SPA of both ear sides were calculated and then included in the analysis. Model 1: The accuracy of speech perception was used as the dependent variable, and time of auditory deprivation, age of cochlear implantation, time of cochlear implantation, and time of rehabilitation training were defined as the independent variables, and linear regression analysis was performed. Model 2: AHT was adjusted and added to model 1. Model 3: Age was adjusted and added to model 2. Because of the significant collinearity between age and age of cochlear implantation (variance inflation factor (VIF) >10), the linear regression calculation of the age of cochlear implantation and SPA was not performed in model 3. *R*^2^ was the goodness of fit, the *t*-test was used to test the regression coefficient, and *p* < 0.05 was considered statistically significant.

## Data Availability

Since the clinical data and personal social background information of cochlear implant patients included in this study involve the privacy of patients. We made a commitment to the patients at the time of enrollment that their clinical data would not be publicly available in any form. So the data is unavailable due to privacy or ethical restrictions.

## References

[1] Niparko J.K., Tobey E.A., Thal D.J., Eisenberg L.S., Wang N.Y., Quittner A.L., Fink N.E. (2010). Team CDI: Spoken Language Development in Children Following Cochlear Implantation. JAMA.

[2] Artieres F., Vieu A., Mondain M., Uziel A., Venail F. (2009). Impact of Early Cochlear Implantation on the Linguistic Development of the Deaf Child. Otol. Neurotol..

[3] Boons T., Brokx J.P., Dhooge I., Frijns J.H., Peeraer L., Vermeulen A., Wouters J., van Wieringen A. (2012). Predictors of spoken language development following pediatric cochlear implantation. Ear Hearth.

[4] Chandrasekaran B., Kraus N. (2010). The scalp-recorded brainstem response to speech: Neural origins and plasticity. Psychophysiology.

[5] Patel A.D., Iversen J.R. (2007). The linguistic benefits of musical abilities. Trends Cogn. Sci..

[6] Niparko J.K. (2004). Speech, language, and reading skills after early cochlear implantation. JAMA.

[7] Svirsky M.A., Teoh S.W., Neuburger H. (2004). Development of language and speech perception in congenitally, profoundly deaf children as a function of age at cochlear implantation. Audiol. Neuro-Otol..

[8] Lee K., Hasselt C.V. (2005). Spoken word recognition in children with cochlear implants: A five-year study on speakers of a tonal language. Ear Hearth.

[9] Rachovitsas D., Psillas G., Chatzigiannakidou V., Triaridis S., Constantinidis J., Vital V. (2012). Speech perception and production in children with inner ear malformations after cochlear implantation. Int. J. Pediatr. Otorhinolaryngol..

[10] Chen Y., Wong L.L.N., Chen F., Xi X. (2014). Tone and sentence perception in young Mandarin-speaking children with cochlear implants. Int. J. Pediatr. Otorhinolaryngol..

[11] Lazard D.S., Vincent C., Venail F., Van de Heyning P., Truy E., Sterkers O., Skarzynski P.H., Skarzynski H., Schauwers K., O’Leary S. (2012). Pre-, Per- and Postoperative Factors Affecting Performance of Postlinguistically Deaf Adults Using Cochlear Implants: A New Conceptual Model over Time. PLoS ONE.

[12] Chen A.T., Li N., Wang Q., Wang M.D., Jiao Q.S., Li S.Y., Li J.N., Yang S.M., Ji F. (2020). Clinical analysis of Chinese Mandarin tone recognition capability in patients with cochlear implantation. Chin. J. Otorhinolaryngol. Skull Base Surg..

[13] Bu X.K., Ni D.F. (2008). To promote the standardization and clinical application of Chinese speech audiometry materials. Chin. J. Otol..

[14] Shi Y., Li Y.S., Wang S.C., Cui D.M., Su Q.T., Wei X.M. (2015). Progress in adult Mandarin speech audiometry materials and their standardization. Int. J. Otolaryngol. Head Neck Surg..

[15] Luo Q., Huang Y.Y., Feng Y.M., Shi H.B. (2016). The Application of Computer-aided Chinese Speech Audiometry Platform to Speech Recognition Text in Children after Cochlear Implantation. Chin. Sci. J. Hearth Speech Rehabil..

[16] Holden L.K., Finley C.C., Firszt J.B., Holden T.A., Brenner C., Potts L.G., Gotter B.D., Vanderhoof S.S., Mispagel K., Heydebrand G. (2013). Factors Affecting Open-Set Word Recognition in Adults with Cochlear Implants. Ear Hearth.

[17] Blamey P., Artieres F., Baskent D., Bergeron F., Beynon A., Burke E., Dillier N., Dowell R., Fraysse B., Gallego S. (2013). Factors Affecting Auditory Performance of Postlinguistically Deaf Adults Using Cochlear Implants: An Update with 2251 Patients. Audiol. Neuro-Otol..

[18] Ji F., Xi X., Chen A.T., Han D.Y. (2007). The effects of scoring unit on monosyllabic word recognition test. Chin. J. Hearth Speech Rehabil..

[19] (2014). Working Guide for Cochlear Implants (2013). Chin. J. Hearth Speech Rehabil..

[20] Zhao J., Zhang H., Ji B., Nizamuddin G. (2010). Diagnostic value of extended high frequency audiometry for early hearing loss in tinnitus patients with normal conventional pure tone audiometry. J. Clin. Otolaryngol. Head Neck Surg..

[21] Cao Y.M., Tao Z.Z., Luo Z.H., Zhu S.Q., Li J., Shu Y., Huang Z.W. (2005). Analysis of high frequency hearing test results in different populations. Chin. J. Hearth Speech Rehabil..

[22] Peng S.C., Tomblin J.B., Cheung H., Lin Y.S., Wang L.S. (2004). Perception and production of mandarin tones in prelingually deaf children with cochlear implants. Ear Hearth.

[23] Ji F., Chen A.T., Zhao Y., Zhou Q.Y., Xi X. (2010). Functional analysis of monosyllabic recognition rate and speech intensity in patients with acoustic neurosis. Chin. J. Hearth Speech Rehabil..

[24] Li J.H., Xi X., Zhao Y., Ji F., Chen A.T., Yang W.Y. (2010). The determination that the maximum speech recognition rate of one syllable decreases disproportionately with pure tone hearing. Chin. J. Otolaryngol. Head Neck Surg..

[25] Wilson B.S., Dorman M.F. (2008). Cochlear implants: Current designs and future possibilities. J. Rehabil. Res. Dev..

[26] Mattys S.L., White L., Melhorn J.F. (2005). Integration of multiple speech segmentation cues: A hierarchical framework. J. Exp. Psychol. Gen..

